# Supplementary material for: Swarming bacteria undergo localized dynamic phase transition to form stress-induced biofilms
Source: eLife. 2021 Mar 16;10:e62632. doi: 10.7554/eLife.62632 (PMC7963483; doi:10.7554/eLife.62632)
Supplement: Transparent reporting form [file elife-62632-transrepform.docx]

***eLife’s* transparent reporting form**

We encourage authors to provide detailed information *within their submission* to facilitate the interpretation and replication of experiments. Authors can upload supporting documentation to indicate the use of appropriate reporting guidelines for health-related research (see [EQUATOR Network](http://www.equator-network.org/%20)), life science research (see the [BioSharing Information Resource](https://biosharing.org/)), or the [ARRIVE guidelines](http://www.plosbiology.org/article/info:doi/10.1371/journal.pbio.1000412) for reporting work involving animal research. Where applicable, authors should refer to any relevant reporting standards documents in this form.

If you have any questions, please consult our Journal Policies and/or contact us: [editorial@elifesciences.org](mailto:editorial@elifesciences.org).

**Sample-size estimation**

- You should state whether an appropriate sample size was computed when the study was being designed
- You should state the statistical method of sample size computation and any required assumptions
- If no explicit power analysis was used, you should describe how you decided what sample (replicate) size (number) to use

Please outline where this information can be found within the submission (e.g., sections or figure legends), or explain why this information doesn’t apply to your submission:

For each of the next experiments, the sample size is:

Wrinkle quantification with 30 µg kan: 47

Wrinkle quantification with blank disk: 22

Wrinkle quantification with eps strain: 12

tasA reporter experiment: 3

control for tasA reporter experiment: 2

island formation visualization experiment in presence of kan: 6

island formation visualization experiment in absence of kan: 6

island formation visualization experiment with a physical barrier: 3

monolayer quantification of cell speed and surface coverage: 32

speed decrease in jam formation: 3

UV experiment to accumulate cells: 18

Split dose of kanamycin experiment 50+150: 20

Split dose of kanamycin experiment 100+100: 22

Split dose of kanamycin experiment 150+50: 19

Split dose of kanamycin experiment 200+0: 19

We consider that the sample size is enough in all the experiments except in ‘control for tasA reporter experiment’ where a 3^rd^ sample will be tested as soon as possible. The information about sample size does not appear in the manuscript because we do not think it is a parameter of interest.

**Replicates**

- You should report how often each experiment was performed
- You should include a definition of biological versus technical replication
- The data obtained should be provided and sufficient information should be provided to indicate the number of independent biological and/or technical replicates
- If you encountered any outliers, you should describe how these were handled
- Criteria for exclusion/inclusion of data should be clearly stated
- High-throughput sequence data should be uploaded before submission, with a private link for reviewers provided (these are available from both GEO and ArrayExpress)

Please outline where this information can be found within the submission (e.g., sections or figure legends), or explain why this information doesn’t apply to your submission:

For each of the next experiments, the number of replicates is:

Wrinkle quantification with 30 µg kan: >20

Wrinkle quantification with blank disk: 5

Wrinkle quantification with eps strain: 3

These three are not reported because we used this data just for illustration.

tasA reporter experiment: 3

control for tasA reporter experiment: 2

These two are reported in the caption of Fig. 2.

island formation visualization experiment in presence of kan: 6

island formation visualization experiment in absence of kan: 6

island formation visualization experiment with a physical barrier: 3

These are not reported because we used this data just for illustration.

monolayer quantification of cell speed and surface coverage: 5

speed decrease in jam formation: 3

UV experiment to accumulate cells: 5

These are not reported because the kind of plots that we decided to use make difficult to plot all the replicates. Therefore, we did not report the number since it does not play any role in the representation of data.

Split dose of kanamycin experiment:5

This last one is not reported but we will introduce it in the caption of the Fig 5 because it is relevant for the plot in fig 5b.

We encountered outliers in the formation of wrinkles. There were periods when wrinkle formation did not happen at all. We discarded all these experiments because we think there is an unknown variable that plays an unknown role in wrinkle formation that we ignore.

**Statistical reporting**

- Statistical analysis methods should be described and justified
- Raw data should be presented in figures whenever informative to do so (typically when N per group is less than 10)
- For each experiment, you should identify the statistical tests used, exact values of N, definitions of center, methods of multiple test correction, and dispersion and precision measures (e.g., mean, median, SD, SEM, confidence intervals; and, for the major substantive results, a measure of effect size (e.g., Pearson's r, Cohen's d)
- Report exact p-values wherever possible alongside the summary statistics and 95% confidence intervals. These should be reported for all key questions and not only when the p-value is less than 0.05.

Please outline where this information can be found within the submission (e.g., sections or figure legends), or explain why this information doesn’t apply to your submission:

No statistical test was done. Fig 2e, Fig 4a,d and 5b have all the raw data plotted. We decided not to plot all the data in Fig 4 b,c by means of clarity. The rest of figures are illustrations.

(For large datasets, or papers with a very large number of statistical tests, you may upload a single table file with tests, Ns, etc., with reference to sections in the manuscript.)

**Group allocation**

- Indicate how samples were allocated into experimental groups (in the case of clinical studies, please specify allocation to treatment method); if randomization was used, please also state if restricted randomization was applied
- Indicate if masking was used during group allocation, data collection and/or data analysis

Please outline where this information can be found within the submission (e.g., sections or figure legends), or explain why this information doesn’t apply to your submission:

Nothing of the stated above was used.

**Additional data files (“source data”)**

- We encourage you to upload relevant additional data files, such as numerical data that are represented as a graph in a figure, or as a summary table
- Where provided, these should be in the most useful format, and they can be uploaded as “Source data” files linked to a main figure or table
- Include model definition files including the full list of parameters used
- Include code used for data analysis (e.g., R, MatLab)
- Avoid stating that data files are “available upon request”

Please indicate the figures or tables for which source data files have been provided:

None. We could provide the codes for the trajectories calculation in the fig 4d.
